# Comparison of growth performance, non-specific immunity, and intestinal microbiota of olive flounder (*Paralichthys olivaceus*) fed with extruded pellet and moist pellet diets under field conditions in South Korea

**DOI:** 10.3389/fmicb.2022.979124

**Published:** 2022-09-02

**Authors:** Won Je Jang, Md. Tawheed Hasan, Wonsuk Choi, Soyeon Hwang, Yein Lee, Sang Woo Hur, Seunghan Lee, Bong-Joo Lee, Youn Hee Choi, Jong Min Lee

**Affiliations:** ^1^Department of Biotechnology, Pukyong National University, Busan, South Korea; ^2^Core-Facility Center for Tissue Regeneration, Dong-Eui University, Busan, South Korea; ^3^Department of Aquaculture, Sylhet Agricultural University, Sylhet, Bangladesh; ^4^Feeds & Foods Nutrition Research Center, Pukyong National University, Busan, South Korea; ^5^Department of Fisheries Biology, Pukyong National University, Busan, South Korea; ^6^Aquafeed Research Center, National Institute of Fisheries Science, Pohang, South Korea; ^7^Department of Smart Fisheries Resources, College of Industrial Sciences, Kongju National University, Yesan, South Korea; ^8^Major in Aquaculture and Applied Life Sciences, Division of Fisheries Life Sciences, Pukyong National University, Busan, South Korea

**Keywords:** extruded pellet, immunity, microbiota, moist pellet, olive flounder

## Abstract

A 6-month feeding trial was conducted to compare the effects of extruded pellet (EP) and moist pellet (MP) feed on the growth performance, non-specific immunity, and intestinal microbiota of olive flounder. A total of 60,000 fish with an average weight of 70.8 ± 6.4 g were divided into two groups and fed with one of two experimental diets. At the end of a 6-month feeding trial, the weight gain and specific growth rate of the fish fed with the MP diets were significantly higher than those of fish fed with EP (*P* < 0.05). However, the EP group exhibited a lower feed conversion rate than the MP group, meaning that the EP diet was more cost-effective. Whole-body proximate compositions and non-specific immune responses (superoxide dismutase, myeloperoxidase, and lysozyme activity) were not significantly different between the two groups. There were no significant differences in the α-diversity of the intestinal bacterial community of the two groups. However, the composition of microorganisms at the phylum to genus level was different between the groups. The EP group was rich in Actinobacteria, *Corynebacterium, Bacillus*, and *Lactobacillus*, whereas the MP group was dominated by Proteobacteria, *Vibrio*, and *Edwardsiella*. Collectively, the MP diet increased growth performance and pathogen concentration in the gut; whereas EP improved feed conversion and beneficial *Bacillus* and *Lactobacillus* proportion in the intestinal microbial community.

## Introduction

The olive flounder (*Paralichthys olivaceus*) is a highly valued fish in East Asian countries such as Korea, Japan, and China. In South Korea, fish, seaweed, and mollusk culture are well-established industries (Hasan et al., 2021) and the technology for *P. olivaceus* aquaculture was introduced from Japan in 1990. However, flounder production in Korea was 17 times greater than that of Japan in 2017 (Sofia, 2018). This demersal carnivore flatfish is the most important aquaculture species in Korea due to its good taste and palatability, high disease resistance, acceptance of different types of feed, and early economic turnover. Feed generally comprises 50–70% of total aquaculture production costs and therefore the nutritional quality, structure, stability, digestion, utilization, and absorption of aquaculture diets are key bottlenecks for the achievement of sustainable commercial fish culture. Currently, flounder diets mostly consist of moist pellets (MP) prepared using frozen raw fish (sardine, herring and mackerel) with locally available binders (Lee et al., 2016).

Over the last two decades, aquaculture scientists have sought to identify low-cost alternatives to fish meal and fish oil from alternative sources such as plants, microbes, seaweeds, and insects (Belghit et al., 2019; Wang et al., 2021). Feed formulation strategies, nutrient stability, leaching in water, and raw material properties are also limiting factors that affect the nutritional profile of supplemented diets, which in turn affects aquaculture profitability (Draganovic et al., 2011). Moreover, fish feeding behavior and digestive tract structure also influence the physical properties of supplemented diets. For example, yellow catfish and snakeheads are surface feeders, whereas rainbow trout and shrimp are bottom feeders and therefore require fast-sinking feeds. Thus, improper feed structure can substantially affect the feeding rates of aquatic species. For flounder, a feeding rate of 11.9% body weight BW/d is considered optimal (Hamidoghli et al., 2020), whereas 13% and 16% feeding rates significantly downregulated the transcription of immune-related genes in this fish species (Lee et al., 2018). Moreover, Sørensen, 2012 reported that pellet extrusion can promote nutrient conservation, water stability (i.e., floatability), and optimum energy content in aquaculture diets.

Extrusion is a complex process that entails the interaction between different ingredients, mass and volume, energy content, momentum, and physiological modulation after cooking, all of which impact the properties of extruded pellet (EP) diets (Samuelsen et al., 2014). Several multinational companies currently produce EP diets for commercial aquaculture ventures. MP diets are also available at the farm level because the availability of local ingredients allows for the preparation of diets at a lower cost and without the need for storage facilities. Olive flounder culture has intensified in Korea; however, this industry has seen a 38% reduction from 2009 to 2017 due to frequent disease outbreaks (Nguafack et al., 2020). The fish intestinal microbiome is a complex ensemble of bacteria, archaea, fungi, and other microorganisms that play key roles in the host's intestinal structure, digestive enzyme secretion, metabolic hemostasis, innate immunity, and disease resistance (Jang et al., 2019, 2021a,b).

There is still a heated debate regarding whether MP and EP diets have different effects on fish production yields, feed digestion and assimilation, nonspecific immunity, microbiome structure, and cost management.

Kim et al. (2014) previously reported significant changes in production, feed utilization, and body protein retention without changes in blood biochemistry, water quality, and histology after feeding olive flounders with MP and EP. Commercial EP and EP diets containing Alaska pollock and herring can replace frozen mackerel-containing MP in terms of production and body composition (Cho et al., 2005). Moreover, formulated EP can replace MP during the growing stage (Kim et al., 2005) and EP increased growth performance, water quality parameters, body protein, and the content of some amino acids relative to MP in *P. olivaceus* culture (Lee et al., 2016). All previous reports have focused on production, feed utilization, blood biochemistry, and body composition; however, no previous studies have evaluated the modulation of innate immunity and intestinal microbiome after dietary supplementation with EP and MP at the farm level.

Therefore, our study sought to evaluate the performance of EP and MP diets in flounder culture at the farm level focusing on non-specific immunity and intestinal microbiota modulation. Moreover, growth performance, body proximate composition, and serum biochemical parameters were also evaluated to understand the effects of these two types of feeds and identify which one is most suitable for farm level applications.

## Materials and methods

### Experimental diet preparation

All experiments were conducted with commercial EP feed (Suhyup feed, South Korea) and MP feed prepared with mackerel and herring. The EP feed had an 8.71% moisture content, 53.8% crude protein, 7.4% crude lipid, and 3.6% crude ash, whereas the MP feed had a 71.1% moisture content, 21.4% crude protein (dry matter basis 59.0%), 2.9% crude lipid (dry matter basis 7.7%), and 1.6% crude ash (dry matter basis 10.6%). Commercially available digestive agents, vitamins C and E, and multivitamins were used as feed additives.

### Fish maintenance and feeding trial

Feeding trials were conducted from April to September (6 months) at an aquaculture farm located in Wando, South Korea. A total of 12 concrete tanks (10 × 10 m) were divided into two groups (EP and MP), and 5,000 fish (average weight of 70.8 ± 6.4 g) were distributed per tank. All of the experimental tanks were flow-through and natural seawater was used during the experiment. Maintenance of the culture system and diet supplementation was performed following the “Rearing and feeding manual of olive flounder” prescribed by the National Institute of Fisheries Science, Republic of Korea.

### Growth performance, feed utilization, and fish body proximate composition

After 6 months of the feeding trial, weight gain, specific growth rate, and feed conversion ratio were calculated as follows: Weight gain (WG; %) = 100 × (Final weight – Initial weight)/Initial weight; Specific growth rate (SGR; %/day) = 100 × (ln final weight – ln initial weight)/days; Feed conversion ratio (FCR) = Dry feed intake/Wet body WG. Fish body proximate composition analysis was performed in accordance with the standard methods of the Association of Official Agricultural Chemists (AOAC (Association of Official Analytical Chemists), 1995).

### Non-specific immune responses and serum biochemical parameters

Serum superoxide dismutase activity (SOD) was determined by calculating the inhibition rate of xanthine oxidase activity using a SOD activity colorimetric assay kit (BioVision) according to the manufacturer's instructions. Serum myeloperoxidase activity (MPO) was estimated as described by Hasan et al. (2018a). Briefly, 80 μl of Hanks' balanced salt solution (without Ca^2+^ or Mg^2+^), 35 μl of 20 mM 3,3′,5,5′-tetramethylbenzidine hydrochloride, and 35 μl of 5 mM H_2_O_2_ were added to 20 μl of serum sample and allowed to react for 2 min. Then, 35 μl of 4 M H_2_SO_4_ was added to stop the reaction. MPO activity was measured at an absorbance of 450 nm. Lysozyme activity was analyzed as described by Hasan et al. (2018a) using *Micrococcus lysodeikticus* cells as a substrate. Briefly, 200 μl of a suspension of 0.75 mg/ml lyophilized *M. lysodeikticus* in PBS was added to 20 μl of serum and allowed to react at room temperature for 30 min. Lysozyme activity was calculated by measuring each absorbance (570 nm) at the initial stage and after 30 min. One unit of lysozyme activity was defined as the amount of serum required to decrease absorbance at 570 nm by 0.001 per minute. Serum glutamic oxaloacetic transaminase (GOT), glutamic-pyruvic transaminase (GPT), total glucose (GLU), and total protein (TP) were measured with a chemical analyzer (Fuji DRI-CHEM 3500i, Fuji Photo Film, Ltd., Japan).

### Intestinal microbiota analysis

After the feeding experiment, the whole intestines were collected by randomly selecting flounder that had been fasted for 24 h and the total DNA of intestinal bacteria was extracted using the FavorPrepTM Tissue Genomic DNA Extraction Mini Kit (Favorgen Biotech Corp., Taiwan). Library construction and sequencing of the V3-V4 regions were conducted at Moagen (Daejeon, Republic of Korea) using an Illumina MiSeq sequencer (300 bp paired-end reads). The obtained raw data were analyzed using the EzBioCloud server (http://www.ezbiocloud.net/).

### Statistical analysis

Normality and homogeneity of variance were assessed for all data using Levene's test. All data were analyzed via Student's t-test using the IBM Statistical Package for the Social Sciences software (SPSS Inc., USA). Statistical significance was determined at a level of *P* < 0.05.

## Results

### Growth performance, feed utilization, and whole-body proximate composition of olive flounders

The final body weight of the MP group (801 g) was significantly higher than that of the EP group (622 g). The weight gain and specific growth rate of fish fed with the MP diet were also significantly higher than those of the fish fed with the EP diet. Moreover, the feed conversion rate was positively modulated in the EP group (1.07) compared to the MP group (3.25) (Table 1). The whole-body proximate composition of olive flounder was not significantly different between the EP and MP groups after 6 months of the feeding trial (Table 2).

**Table 1:** Growth performance and feed utilization of olive flounder fed with the experimental diets for 6 months^1^.

	**Extruded pellet**	**Moist pellet**	**Pooled SEM^2^**
IBW (g)^3^	69.4	71.9	1.3
FBW (g)^4^	622^a^	801^b^	89.5
WG (%)^5^	796.25^a^	1014.05^b^	108.9
SGR (%/day)^6^	1.42^a^	1.65^b^	0.1
FCR^7^	1.07^a^	3.25^b^	1.1

1 Values are means of five replicate groups of fish. Values with different superscript letters within the same row in the table are significantly different (*P* < 0.05). The lack of superscript letter indicates no significant differences (*P* > 0.05).

2 Pooled SEM: standard deviation/√n.

3 IBW (g): initial body weight = initial body weight of total fish/initial fish number.

4 FBW (g): final body weight = final body weight of total fish/final fish number.

5 WG (%): weight gain = [(final weight–initial weight)/initial weight] × 100.

6 SGR(%/day): specific growth rate = [(ln final weight–ln initial weight)/days] × 100.

7 FCR: feed conversion ratio = dry feed intake/wet body weight gain.

**Table 2:** Whole-body proximate compositions of olive flounder fed with the experimental diets for 6 months^1^.

	**Extruded pellet**	**Moist pellet**	**Pooled SEM^2^**
Moisture (%)	71.5	70.9	0.3
Crude Ash (%)	4.3	4.4	0.1
Crude Protein (%)	18.1	18.1	0.0
Crude Lipid (%)	3.8	4.0	0.1

1 The values represent the pooled means of 9 fish per diet. All values within the same row in the table are not significantly different (*P* > 0.05, *n* = 6).

2 Pooled SEM: standard deviation/√n.

### Non-specific immune responses and serum biochemical parameters

SOD, MPO, and LYZ, which were analyzed as non-specific immune parameters, were not significantly different between the two groups (Table 3). GOT, GPT, and TP, which were analyzed as serum biochemical parameters, also exhibited no significant differences between the two groups. The GLU values were 13.3 in the EP group and 18.0 in the MP group, indicating a significant difference between the groups (Table 4).

**Table 3:** Non-specific immune responses in olive flounder fed with the experimental diets for 6 months^1^.

	**Extruded pellet**	**Moist pellet**	**Pooled SEM^2^**
SOD^3^	38.3	47.6	4.6
MPO^4^	1.6	1.8	0.1
LYZ^5^	0.1	0.2	0.1

1 The values represent the pooled means of 9 fish per diet. All values within the same row in the table are not significantly different (*P* > 0.05, *n* = 6).

2 Pooled SEM: standard deviation/√n.

3 SOD: superoxide dismutase activity (% inhibition).

4 MPO: myeloperoxidase activity (O.D. at 450 nm).

5 LYZ: lysozyme activity (U/ml).

**Table 4:** Serum biochemical parameters in olive flounder fed with the experimental diets for 6 months^1^.

	**Extruded pellet**	**Moist pellet**	**Pooled SEM^2^**
GOT (U/L)^3^	17.0	18.7	0.9
GPT (U/L)^4^	5.7	7.3	0.8
GLU (mg/dl)^5^	13.3^a^	18.0^b^	2.4
TP (g/dl)^6^	4.4	5.9	0.7

1 The values represent the pooled means of 9 fish per diet. All values within the same row in the table are not significantly different (*P* > 0.05, *n* = 6).

2 Pooled SEM: standard deviation/√n.

3 GOT: glutamic oxaloacetic transaminase.

4 GPT: glutamlc pyruvic transamlnase.

5 GLU: total glucose.

6 TP: total protein.

### Intestinal microbiota analysis

The α-diversity of the analyzed olive flounder intestinal bacterial communities was not significantly different between the two groups. The number of operational taxonomic units (OTUs) found in the EP and MP groups were 220 ± 69 and 220 ± 52, respectively. The ACE, CHAO, and Jackknife richness estimates of the EP group were 243 ± 76, 236 ± 75, and 248 ± 79, whereas those of the MP group were 244 ± 64, 234 ± 60, and 251 ± 64, respectively. The Shannon and Simpson values for diversity estimation in the EP group were 3.42 ± 0.43 and 0.09 ± 0.05, whereas those in the MP group were 2.92 ± 0.53 and 0.12 ± 0.07, respectively (Table 5). These results indicated that the α-diversity of the intestinal bacterial community of olive flounder was not altered by the EP and MP diets.

**Table 5:** α-Diversity of the intestinal bacterial communities of olive flounder^1^.

**Groups**	**OTUs**	**ACE**	**CHAO**	**Jackknife**	**Shannon**	**Simpson**
Extruded pellet	220 ± 69	243 ± 76	236 ± 75	248 ± 79	3.42 ± 0.43	0.09 ± 0.05
Moist pellet	220 ± 52	244 ± 64	234 ± 60	251 ± 64	2.92 ± 0.53	0.12 ± 0.07

1 Values are mean ± SD of five replicates. All values within the same column in the table are not significantly different (*P* > 0.05, *n* = 5).

**Figure 1:**
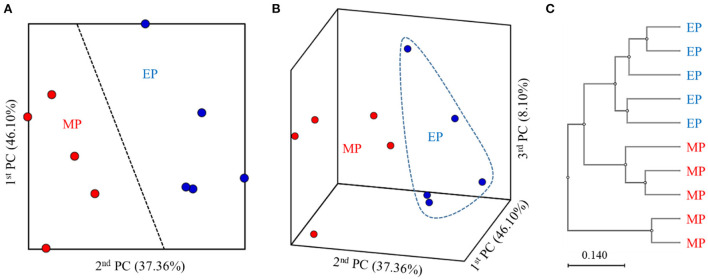
2D **(A)** and 3D **(B)** principal coordinate analysis based on the weighted UniFrac metrics and unweighted pair group method with arithmetic mean tree **(C)** of bacterial operational taxonomic units between the different diets.

2D and 3D analysis of the β-diversity at the genus level based on the weighted UniFrac metrics using principal coordinate analysis showed clear boundaries between the groups (Figures 1A,B). Moreover, the unweighted pair group method with arithmetic mean tree analysis elucidated clear differences between the groups (Figure 1C).

**Figure 2:**
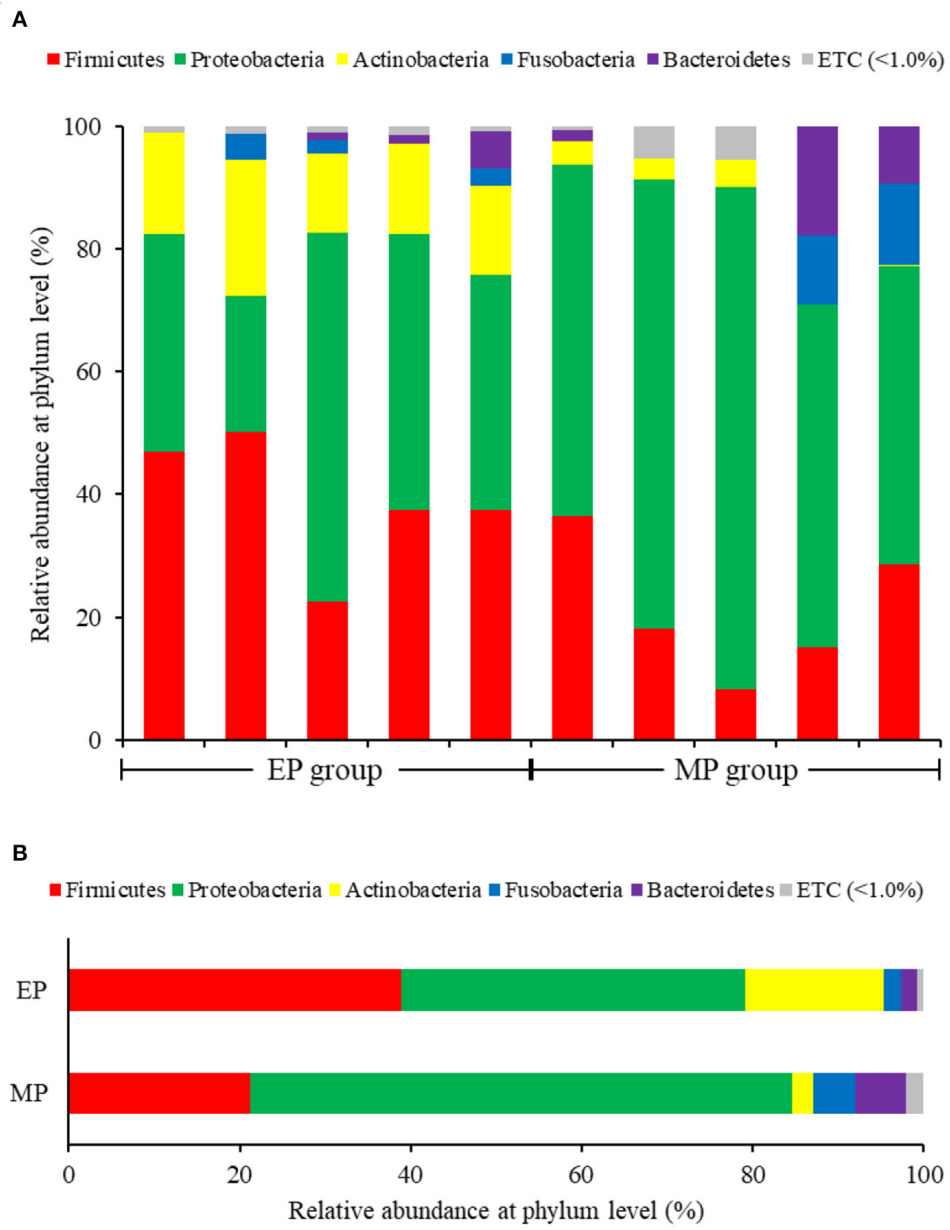
Individual **(A)** and average **(B)** composition and relative abundance of intestinal bacterial communities of olive flounder fed with different diets at the phylum level.

In the analysis of the composition and relative abundance of intestinal bacterial communities at the phylum level, Proteobacteria were the most abundant in both groups, followed by Firmicutes. However, Proteobacteria were more abundant in the MP group (63.40%) than in the EP group (40.20%). Furthermore, Firmicutes were more abundant in the EP group (38.92%) than in the MP group (21.29%). Actinobacteria were relatively abundant only in the EP group (16.23%; MP group: 2.43%) (Figure 2).

**Figure 3:**
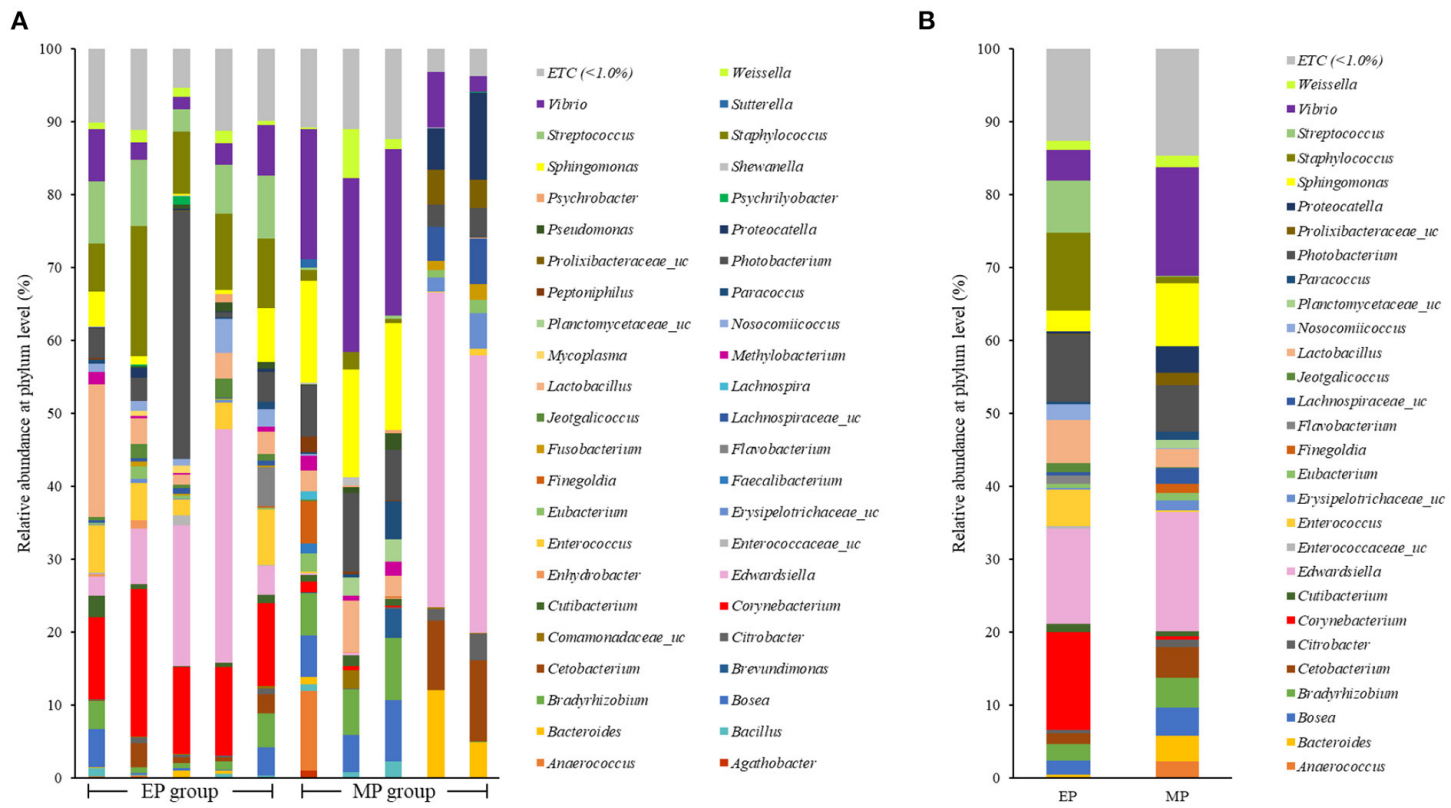
Individual **(A)** and average **(B)** composition and relative abundance of intestinal bacterial communities of olive flounder fed with different diets at the genus level.

Figure 3 shows the intestinal microbial composition of olive flounder at the genus level. Corynebacterium was the most abundant genus in the EP group, and Lactobacillus and Bacillus were also present at a higher ratio in the EP group than in the MP group. The most abundant genus in the MP group was Edwardsiella, followed by Vibrio. Bacillus, the most studied probiotic of olive flounder, was present in proportions below 1.0% in both the EP (0.75%) and MP (0.40%) groups.

**Figure 4:**
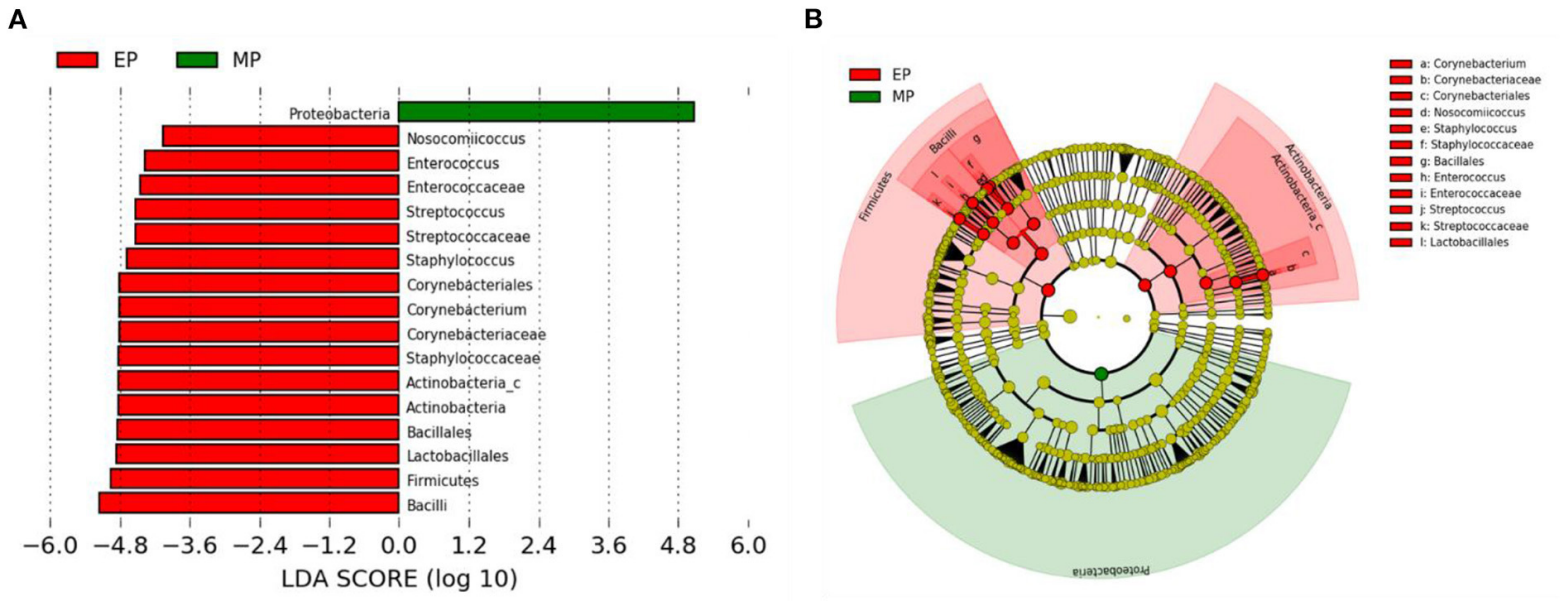
Linear discriminant analysis effect size (LEfSe) analysis of the differential abundance of taxa within olive flounder intestinal microbiota following random sampling from each group. **(A)** Linear discriminant analysis (LDA) score of the abundance of taxa. **(B)** Cladogram showing differentially abundant taxa between the two groups from phylum to genus.

Linear discriminant analysis (LDA) effect size (LEfSe) analysis showed that Corynebacterium and Bacilli, including subgroups such as Lactobacillales, Bacillales, Staphylococccaceae, and Streptococccaceae, were abundant in the EP group, whereas Proteobacteria was more abundant in the MP group (Figure 4).

**Figure 5:**
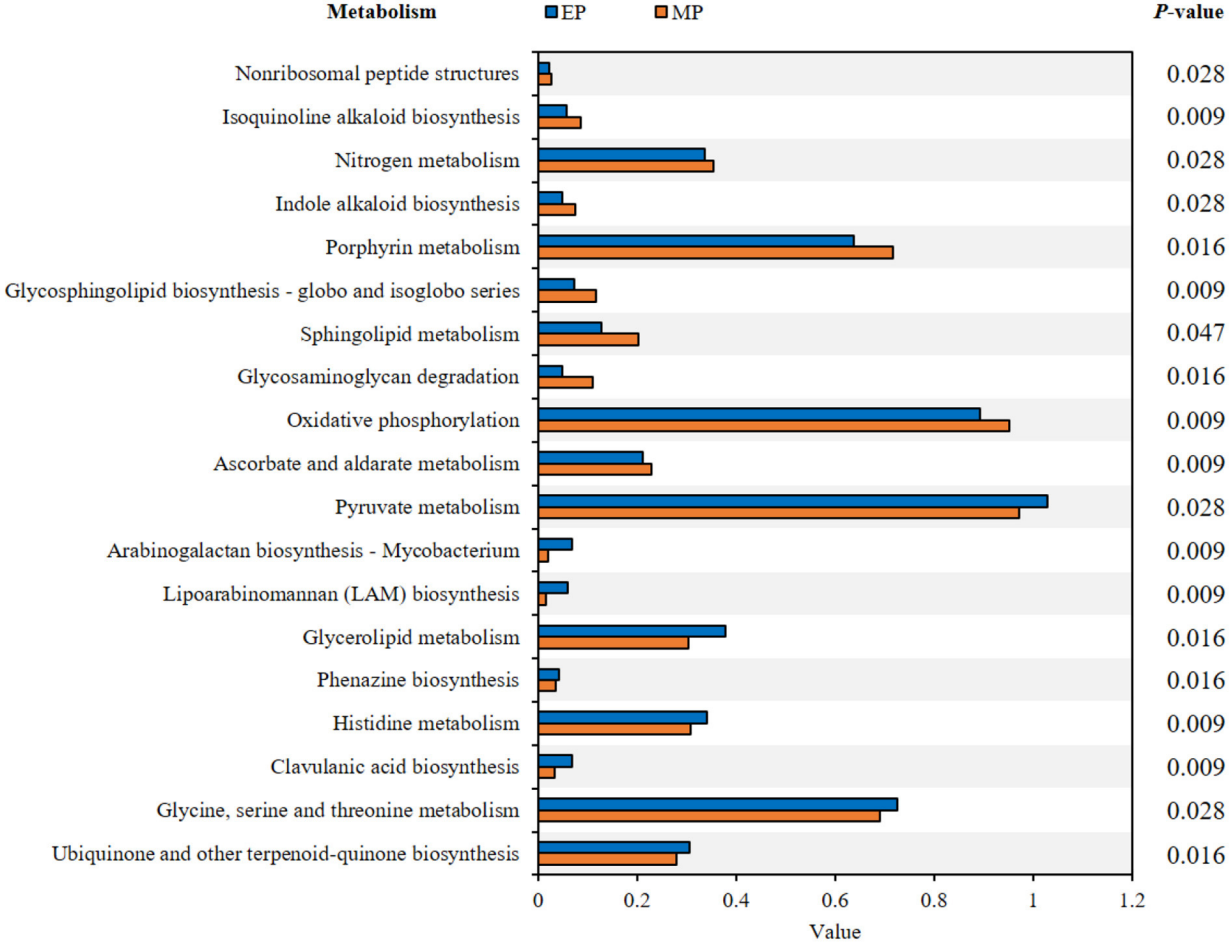
Presumptive metabolism functions of intestinal microbiota in olive flounder with different diets. Kyoto Encyclopedia of Genes and Genomes (KEGG) pathways were obtained from microbiota analysis data using PICRUSt.

The presumptive metabolism functions of the intestinal microbiota of olive flounder fed with the experimental diets for 6 months were compared based on metagenome data using PICRUSt. The functional pathways leading to significant differences are detailed in Figure 5. Compared to the MP group, 9 pathways were significantly enriched in the EP group including the pyruvate metabolism pathway, whereas 10 pathways were significantly enriched in the MP group in including the ascorbate and aldarate metabolism pathway (Figure 5).

## Discussion

Aquaculture is one of the fastest growing food production sectors worldwide (FAO, 2020). Seafood constitutes a high-quality protein source and therefore its production must continue to increase to ensure food security, particularly in developing countries (de Godoy et al., 2022). However, concerns regarding the environmental impact of food production systems have increased in recent decades (de Godoy et al., 2022), and therefore maintaining a farm environment for sustainable and healthy aquaculture production with low environmental pollution has become critical. One of the key challenges faced by the aquaculture industry entails the development of strategies to overcome wasteful production systems, including fish overstocking, overfeeding, and excessive use of drugs (e.g., antibiotics) and other therapeutic agents (Song, 2011). These wasteful practices not only lead to environmental pollution but also increase the incidence of fish diseases, increase mortality rates, and decrease water quality (Cho et al., 2009; Song, 2011). Therefore, research on environmental conservation and establishing sustainable food production systems are essential for the future development of aquaculture.

The substitution of MP feed for EP feed is a priority for sustainable aquaculture. Song (2011) reported that MP and EP diets did not affect the survival rate of olive flounder but did affect its growth rate. In the present study, the MP feed promoted fish growth. However, this diet had very poor feed efficiencies, which translates to large economic losses. Additionally, the lost feed accumulates on the bottom of the water body and decomposes, thus aggravating marine pollution and deteriorating the aquaculture environment (Kim et al., 2007). Additionally, an increase in the price of fish, a major component of MP feed, may have a negative impact on profitability. In contrast, EP feed showed very positive feed efficiency. EP feed is cheaper than MP feed, which would make flounder production more resilient to price fluctuations. MP feed is more expensive, requires more labor to produce, and causes various diseases and environmental pollution, but it is still preferred over EP feed because it delivers high growth rates, thus guaranteeing high yields. However, EP feed is a more environmentally friendly and sustainable alternative. Therefore, further research and development should compensate for the limitations of EP feed. For example, new additives could be developed to aid feed digestion and absorption, as well as to stimulate appetite to increase fish growth rates, all of which would greatly promote the use of EP feeds.

Similar to terrestrial vertebrates, fish possess innate immunity mechanisms such as SOD, lysozyme, and MPO, which act as the first line of defense against the spread and movement of external pathogens in the body (Hasan et al., 2018a; Jang et al., 2021a). SOD is an enzyme that maintains immunological balance and prevents tissue damage, MPO contributes to common inflammatory responses such as inducing neutrophil antimicrobial response and stimulating macrophages, and LYZ is the cornerstone of nonspecific immunity to destroy pathogens (Castro et al., 2008; Ragland and Criss, 2017; Hoseinifar et al., 2020). Moreover, in the antioxidant defense pathway, highly reactive ${\text{O}}_{2}^{-}$ is catalyzed and converted to less reactive H_2_O_2_ and finally to water (H_2_O) by the SOD enzyme activity (Hasan et al., 2018b). In this study, no significant difference was found in the non-specific immune parameters of the MP and EP groups, indicating that the MP and EP diets did not affect the non-specific immune system of olive flounder. Hematological characteristics can be used as indicators of the nutrition, health, and stress status of fish (Lee et al., 2016). According to Wells et al. (1986), plasma GOT and GPT activity can provide information on liver damage or dysfunction and is therefore used as a stress indicator in a variety of fish species (Almeida et al., 2002; Lee et al., 2016). In this study, there was no significant difference in GOT and GPT values between the experimental groups, suggesting that there was no difference in stress due to liver injury or dysfunction. However, plasma glucose, a key stress indicator in fish, was significantly higher in the MP group than in the EP group. Lee et al. (2016) proposed that these changes could be due to the stress of feed competition and could be higher in the group of fish fed with the MP diet than the fish fed with the EP diet.

The gut of aquatic animals contains a large number of microorganisms, which are altered by a variety of factors such as the growth stage of fish, feeding activity, host species, rearing conditions, habitat, and water quality (Jang et al., 2021a; Gao et al., 2022). The microbiome affects the nutritional, physiological, and immunological functions of the host, thus ultimately affecting their growth and development (Ni et al., 2014; Gao et al., 2022). Therefore, imbalances in the gut microbiota can lead to common metabolic diseases including fatty liver, diabetes, oxidative damage, and inflammatory responses, which negatively affect the growth and development of fish. In contrast, the modulation of the gut microbiota with supplements such as probiotics can be an excellent strategy to increase fish growth and immunity in aquaculture (Cicala et al., 2020; Fan and Pedersen, 2021; Jang et al., 2021a; Wastyk et al., 2021; Bruni et al., 2022; Gao et al., 2022). Several studies have explored the effects of growth stage, probiotic supplementation, and feed ingredients on the intestinal microbiota of fish. However, to the best of our knowledge, no previous studies have compared the intestinal microbiota of olive flounder fed with MP and EP feed in field conditions (Jang et al., 2019, 2021a,b; Niu et al., 2019, 2020).

α-Diversity is an important indicator of the homeostasis of intestinal microbiota (Shi et al., 2022). The ACE and CHAO indices measure richness, whereas the Shannon and Simpson indices measure diversity. In this study, no significant differences were found between the α-diversity of the intestinal microbiota of olive flounder fed with the MP and EP diets for 6 months under field conditions. This suggests that the MP and EP diets did not affect the intestinal microbial richness and evenness of olive flounder. The β-diversity analysis of the gut microbiota can identify similarities in the community structure of various samples (Zeng et al., 2020). Analysis with weighted UniFrac metrics showed that the differences within each sample group were small and all could be grouped into clusters. The distribution distances between groups were relatively long, suggesting that there are certain differences between MP- and EP-fed groups with respect to bacterial community structure.

Firmicutes and Bacteroidetes are the most abundant phylum in most vertebrates, including amphibians, reptiles, mammals, and birds (Hong et al., 2011; Human Microbiome Project Consortium., 2012; Waite and Taylor, 2014; Kim et al., 2021). In contrast, the fish gut microbiota is dominated by Proteobacteria and Firmicutes (Kim et al., 2021). As with other fish, the dominant phyla in the olive flounder investigated in this study were Proteobacteria and Firmicutes. According to our findings, the EP group had a higher proportion of Firmicutes than the MP group. Representative genera within the Firmicutes phylum include Bacillus, Lactobacillus, and Enterococcus. These are well known as probiotics, and previous studies have reported that they promoted the growth and immunity of olive flounder (Hasan et al., 2018a, 2021; Jang et al., 2019, 2021a,b). The EP group also exhibited a relatively high abundance of the phylum Actinobacteria. Ramírez and Romero, 2017 reported that Actinobacteria are abundant in wild flounder, and some of the bacteria belonging to this phylum produce antimicrobial compounds and valuable substances such as amino acids, vitamins, enzymes, specific growth factors, pigments, and polysaccharides.

Assessing the bacterial community of each group provides information about the relationship between this community and the host, which can be associated with the functions that a particular bacterial genus can provide within the host (Ramírez and Romero, 2017). In the EP group, 9 functional pathways related to carbohydrate, lipid, and amino acid metabolism, biosynthesis of other secondary metabolites, glycan biosynthesis and metabolism, and metabolism of cofactors and vitamins were found to be significantly enriched compared to the MP group. The functional pathways enriched in the MP group were related to energy, carbohydrate, and lipid metabolism, biosynthesis of other secondary metabolites, glycan biosynthesis and metabolism, metabolism of terpenoids and polyketides, and metabolism of cofactors and vitamins. However, given that functional pathway analysis is predicted based on the bacterial community, further analysis is required to identify the function of intestinal microorganisms.

The development of EP diets is essential for the sustainable and healthy production of aquaculture. The administration of EP diets in field conditions enhanced the feed conversion rates of olive flounder but showed a lower growth rate compared to the conventional MP diet. The EP and MP diets did not significantly affect non-specific immune responses and biochemical parameters. Moreover, gut microbiota analysis showed that the EP diet increased the abundance of beneficial bacteria such as *Bacillus* and *Lactobacillus*. In conclusion, the use of EP feed is recommended, however, additives and appetite stimulants must be developed to increase the growth rate of fish fed with EP feeds in order to completely replace MP feed.

## Data availability statement

The original contributions presented in the study are included in the article/supplementary materials, further inquiries can be directed to the corresponding author.

## Ethics statement

The animal study was reviewed and approved by the study was conducted under the guidelines of the Animal Ethics Committee Regulations of Pukyong National University.

## Author contributions

WJJ: methodology, software, investigation, data curation, and writing—original draft. MTH: software, data curation, and writing—original draft. WC, SH, and YL: data curation and investigation. SWH, SL, and B-JL: conceptualization and resources. YHC: supervision and project administration. JML: data curation, supervision, and writing—review and editing. All authors contributed to the article and approved the submitted version.

## Funding

This work was financially supported by the grant (R2022016) from the National Institute of Fisheries Science, Republic of Korea and Basic Science Research Program through the National Research Foundation of Korea (NRF) funded by the Ministry of Education (2021R1I1A1A01049238).

## Conflict of interest

The authors declare that the research was conducted in the absence of any commercial or financial relationships that could be construed as a potential conflict of interest.

## Publisher's note

All claims expressed in this article are solely those of the authors and do not necessarily represent those of their affiliated organizations, or those of the publisher, the editors and the reviewers. Any product that may be evaluated in this article, or claim that may be made by its manufacturer, is not guaranteed or endorsed by the publisher.
